# Soil–Arthropod Interactions Across Forest Types in a Mediterranean Mountain Landscape

**DOI:** 10.3390/insects17100988

**Published:** 2026-09-24

**Authors:** María Villa, Diogo Angélico, Marina Castro

**Affiliations:** CIMO, LA SusTEC, Universidade Politécnica de Bragança, Campus de Santa Apolónia, 5300-253 Bragança, Portugal; diogoangelico@unipb.pt (D.A.); marina.castro@unipb.pt (M.C.)

**Keywords:** ground-dwelling arthropods, edaphic gradients, functional groups, seasonal variation, taxonomic composition, Mediterranean mountain forests

## Abstract

Ground-dwelling arthropods contribute to decomposition, nutrient cycling, and biological control, but their relationships with soil properties remain insufficiently understood in Mediterranean mountain forests. We evaluated taxonomic and functional structure, seasonal variation, and associations with edaphic gradients across three forest stands—one holm oak (*Quercus rotundifolia*), one Pyrenean oak (*Quercus pyrenaica*), and one maritime pine (*Pinus pinaster*)—in Montesinho Natural Park, northeastern Portugal. Arthropods were sampled with pitfall traps during four campaigns from July 2023 to April 2024, and soil properties were characterized at each trap. We recorded 5511 arthropods, comprising 18 orders, 54 taxa, and six functional groups. Total abundance was lowest in February, highest in April, and lower in the Pyrenean oak stand than in the other stands. Taxon richness, predator abundance, and the abundance of taxa with unresolved or multiple trophic functions varied differently among stands across campaigns. The sampling campaign explained 28.7% of the variation in taxonomic composition. Total abundance and omnivore abundance, the latter dominated by Formicidae, increased along PC1, associated with finer-textured soils, higher pH and C:N ratio, and lower available potassium. Omnivores decreased along PC2, associated mainly with organic matter, roots, and clay-bound nitrogen. PC1 and PC2 jointly explained 33.2% of the variation in taxonomic composition. Overall, the results show that seasonal and edaphic variation are important in structuring the taxonomic and functional components of ground-dwelling arthropod communities in Mediterranean mountain forests.

## Simple Summary

Arthropods living and moving on the soil surface contribute to important forest processes, including decomposition, nutrient recycling, and the regulation of other invertebrates. We studied these organisms in holm oak, Pyrenean oak, and maritime pine forests in Montesinho Natural Park, northeastern Portugal. Arthropods were collected during summer, autumn, winter, and spring, and several soil characteristics were measured at the same sampling points. Arthropod abundance was lowest in winter and highest in spring, although seasonal changes were not the same in the three forests. The taxa contributing to ecological groups such as predators and omnivores also changed among seasons and forests. Soil gradients related to texture, acidity, organic matter, roots, nitrogen, and potassium were associated with arthropod abundance and community composition. Ants showed particularly strong seasonal and soil associations, while predator taxa changed markedly among seasons and forests. Bark beetles accounted for the spring increase in plant-feeding arthropods in the pine forest, and flies and mites made a major contribution to overall abundance. These findings identify several informative groups for future monitoring and improve our understanding of how forest arthropod communities vary throughout the year and in relation to soil conditions in Mediterranean mountain landscapes.

## 1. Introduction

Ground-dwelling arthropods are an essential component of biodiversity and contribute to multiple ecological processes. These organisms participate in organic matter decomposition and nutrient cycling, regulate other invertebrate populations, influence soil characteristics, contribute to seed dispersal, and connect above- and below-ground food webs. Overall, they play an essential role in forest ecosystem functioning [1,2]. They also respond to environmental variation, serving as indicators of habitat disturbance and forest management [3,4].

Arthropod communities can be examined using complementary taxonomic and functional approaches. Taxonomic analyses reveal changes in the identity and relative abundance of taxonomic categories, whereas functional analyses provide information on their potential ecological roles. In Iberian wood-pastures, small shrubby patches supported functionally distinct assemblages of plants, beetles, and lichens and contributed to habitat-scale functional diversity [5]. In Iberian holm oak savannas, the duration of livestock exclusion was associated with differences in arthropod taxonomic diversity and community composition, whereas functional diversity was closely related to vegetation structure [6]. Taxonomic and functional responses may also differ between arthropod groups. For example, epigeic ant and spider communities showed contrasting responses to wildfire over a four-year period, and their taxonomic and functional diversities did not always respond in the same way [7].

The structure of ground-dwelling arthropod communities depends on several factors, including vegetation, soil, and microclimatic conditions, which interact with each other. Forest vegetation and structure influence aspects such as understory vegetation, litter quantity and quality, deadwood structure, and resource availability. These characteristics modify temperature and moisture conditions at the soil surface and provide food and shelter for different arthropod groups [1,4]. In Mediterranean wood pastures of north-western Spain, differences in soil fertility, litter accumulation, and microhabitat characteristics were associated with the abundance and composition of several ground-dwelling arthropod groups [8]. Within the Montesinho Natural Park, plant taxonomic and functional diversity was also positively associated with the abundance of several arthropod functional groups during spring, supporting the relevance of bottom-up plant–arthropod relationships in this mountain landscape [9].

Several soil properties, including texture, organic matter content, pH, nutrient availability, and the carbon-to-nitrogen ratio, have been associated with arthropod abundance and community structure [10,11,12]. These properties may influence water availability, microhabitat conditions, and the resources supporting soil food webs. Soils with larger proportions of fine particles generally have a greater water-retention capacity, although this also depends on soil structure, porosity, and organic matter content [13]. Soil texture has been associated with variation in ground-active arthropod abundance, but its effects may depend on vegetation and microhabitat conditions [12].

Changes in microclimatic conditions and resource availability across seasons can affect arthropod communities by altering vital processes such as mobility, feeding activity, reproduction, and trophic interactions. For example, in a mature oak woodland sampled throughout a complete annual cycle, arthropod abundance and diversity were found to generally increase from spring to summer, with temperature being the main environmental predictor of pitfall and Malaise trap captures [14]. Forest structure can also modify temperature heterogeneity within stands. In temperate forests, season, vertical position, and temperature were associated with arthropod community composition across forest microhabitats [15]. In a Mediterranean agroforestry system, sampling date was the main factor associated with arthropod community composition, whereas the effects of agroforestry on abundance and diversity differed among eight trophic groups [16].

These seasonal relationships may be particularly important in transitional Mediterranean–sub-Atlantic environments, where arthropod communities experience both summer water stress and low winter temperatures. For example, within the Montesinho Natural Park, a transitional Mediterranean–sub-Atlantic region, ant communities differed in taxonomic and functional composition between oak forests and chestnut orchards, and their seasonal dynamics depended on habitat conditions [17].

Montesinho Natural Park, in north-eastern Portugal, comprises a heterogeneous landscape that includes native forests dominated by *Quercus pyrenaica* Willd. and *Quercus rotundifolia* Lam. (Fagaceae), as well as plantations of *Pinus pinaster* Aiton (Pinaceae). Previous research in the park has examined relationships between plant diversity and arthropod functional groups during spring [9] and habitat-dependent taxonomic, functional and seasonal variation in ant communities [17]. In other regions of the Iberian Peninsula, studies have related soil fertility, litter accumulation and microhabitat structure to several ground-dwelling arthropod groups [8], or examined temporal taxonomic and functional responses in selected epigeic taxa [7]. However, few studies have jointly examined ground-dwelling arthropod communities over a complete annual cycle by integrating taxonomic composition, functional groups, and multivariate soil gradients. Such an integrated approach can help determine how edaphic conditions are associated with community structure and with the ecological functions potentially represented by different arthropod groups.

In this study, the goal was to evaluate variation in ground-dwelling arthropod communities in *Q. pyrenaica*, *Q. rotundifolia*, and *P. pinaster* stands over an annual cycle, as well as the relationships of these communities with soil characteristics. Specifically, we studied: (i) taxonomic and functional community structure; (ii) variation in total abundance, taxon richness, and functional-group abundance among stands and throughout the annual cycle; (iii) temporal variation in taxonomic composition; and (iv) associations between arthropod communities and soil characteristics. We hypothesized that (i) arthropod abundance and taxon richness would be lower in winter and higher in spring; (ii) community attributes and their seasonal variation would differ among the three stands; and (iii) taxonomic and functional components would show different associations with the edaphic gradients.

## 2. Materials and Methods

### 2.1. Study Area

The study was conducted in the central sector of Montesinho Natural Park (northeastern Portugal), within the parishes of Parâmio and Espinhosela. Three forest habitats representative of the region were selected: a Pyrenean oak (*Quercus pyrenaica* Willd.) woodland, a holm oak (*Quercus rotundifolia* Lam.) woodland, and a maritime pine (*Pinus pinaster* Aiton) plantation (Figure 1).

The *Q. pyrenaica* stand is located near the village of Zeive (41°54.513′ N, 6°53.696′ W) and covers approximately 2.4 ha at elevations ranging from 895 to 934 m a.s.l. It has an estimated tree density of approximately 1545 trees ha^−1^ and shrub cover of approximately 32% [18]. The understory was dominated by shrubs of the genera *Erica*, *Genista*, *Rubus*, *Rosa*, and *Lavandula*, together with abundant herbaceous species, particularly *Brachypodium rupestre* and *Pteridium aquilinum*. The soil is predominantly classified as an Umbric Leptosol and is generally shallow, gravelly, and sandy loam in texture [18]. The stand was approximately 2600 m from the Baceiro River.

The *Q. rotundifolia* woodland and the *P. pinaster* plantation are located near the village of Vilarinho (41°54.016′ N, 6°50.872′ W), approximately 4 km east of the *Q. pyrenaica* site. The holm oak stand covers approximately 2.7 ha at elevations between 804 and 826 m a.s.l. It has an estimated tree density of approximately 965 trees ha^−1^ and shrub cover of approximately 51% [18]. Its understory was dominated by shrubs of the genera *Cistus*, *Cytisus*, and *Lavandula*, with *Anthoxanthum aristatum* as the dominant herbaceous species. The soil is classified as a Leptosol developed over basic rocks and is generally shallow, gravelly, and sandy loam in texture [18]. The stand was approximately 286 m from the Baceiro River.

The maritime pine plantation occupies approximately 1.2 ha at elevations between 806 and 821 m a.s.l. According to local management information, it was planted approximately 45–50 years before the present study. In 2011–2012, a sanitary intervention removed trees affected by pine wilt disease. At the time of sampling, it was a mature stand with a sparse understory and limited natural regeneration. No regular understory clearing had been required in recent years. The stand was approximately 232 m from the Baceiro River.

According to the bioclimatic classification of Monteiro-Henriques et al. [19], all study sites belong to the lower humid supramediterranean belt. The climate is characterized by a mean annual precipitation of 1000–1200 mm, a mean annual temperature of 11–13 °C, mean annual maximum temperatures of 15–17 °C, and mean annual minimum temperatures of 4–7 °C, based on the 1981–2010 climatic normals provided by the Portuguese Institute for Sea and Atmosphere (IPMA) [20].

### 2.2. Experimental Design

Ten sampling points were randomly established in each oak stand, whereas five were established in the pine stand because of its smaller area (1.2 ha, compared with 2.4–2.7 ha for the oak stands). Sampling points were separated by at least 20 m to ensure spatial independence. At each sampling point, ground-dwelling arthropods and soil samples were collected. The study included one stand of each forest type; consequently, comparisons concern these three specific stands rather than replicated effects of forest type.

### 2.3. Arthropod Sampling

Ground-dwelling arthropods were sampled during four campaigns conducted from 21 July 2023 to 28 July 2023, 24 October 2023 to 31 October 2023, 31 January 2024 to 7 February 2024, and 23 April 2024 to 30 April 2024, corresponding to July, October, February, and April, respectively. Traps remained active for seven consecutive days during each sampling period.

Arthropods were collected using one pitfall trap per sampling point (Figure 1). Each trap consisted of a plastic cup (7 cm diameter × 9.5 cm depth) installed flush with the soil surface. Each trap was filled with approximately 150 mL of a 3:1 water–propylene glycol solution, with three to four drops of detergent added to reduce surface tension. Propylene glycol acted as a preservative and prevented the solution from freezing during winter sampling. Traps were protected with a plastic roof (12.5 cm diameter) positioned approximately 2 cm above the soil surface to minimize rainwater entry and reduce the accidental capture of small vertebrates. Collected samples were transported to the laboratory, where arthropods were separated from debris and the preservative solution by filtration and subsequently preserved in 70% ethanol until processing. Specimens were sorted and quantified under a Leica MicroSystems Microscopes, EZ4 Fixed 10x 10447197, Maia, Portugal and identified to the lowest possible taxonomic level. Orders and families were identified using Triplehorn and Johnson [21], while Coleoptera were additionally identified using Albouy and Richard [22].

Each taxon was assigned to one of six broad functional categories according to its predominant or potential trophic ecology: predators, decomposers, phytophagous, parasitoids, omnivores, and taxa with unresolved or multiple trophic functions. The category “taxa with unresolved or multiple trophic functions” included taxa that could not be assigned reliably to a single predominant function because they were identified only at a broad taxonomic level or encompassed markedly different feeding strategies. These categories represent broad trophic assignments and should not be interpreted as mutually exclusive functional guilds.

Mites were identified only at the level of Acari and were not separated into lower taxonomic groups. Because Acari includes groups occupying different trophic positions, they were assigned to the category “taxa with unresolved or multiple trophic functions”.

Collembola were excluded from the dataset because their very high abundance and the associated processing constraints prevented consistent quantification across all samples.

Pitfall catches were interpreted as measures of activity-density at the soil surface rather than absolute population density. Winged arthropods captured when landing on or moving across the forest floor were retained in the taxonomic description. However, Diptera and other predominantly flying taxa are sampled less consistently by pitfall traps, and their recorded abundance should therefore be interpreted with caution.

The specimens are preserved in 70% ethanol at the Terrestrial Ecology Laboratory of the Polytechnic Institute of Bragança, Bragança, Portugal, under the responsibility of María Villa.

### 2.4. Soil Sampling and Analysis

A total of 25 disturbed soil samples were collected between June and July 2023 from the 0–10 cm soil layer at the sampling points using an open cylindrical soil auger to determine soil pH (H_2_O), organic matter (OM), available phosphorus (P), available potassium (K), total calcium carbonate (CaCO_3_), total carbon (TC), total nitrogen (TN), and carbon and nitrogen associated with the sand, silt, and clay fractions. Additionally, 25 undisturbed soil cores were collected from all three habitats in January 2025 at depths of 0–5 cm and 5–10 cm using 100 cm^3^ steel cylinders to determine soil bulk density. Because the study sites were unmanaged forest stands and no management interventions or major disturbances occurred during the study period, soil physicochemical properties were determined once for each sampling point and used in all subsequent analyses. Accordingly, the same soil physicochemical properties were assigned to all arthropod sampling dates for each sampling point.

All soil samples were transported to the laboratory for processing. Disturbed soil samples were oven-dried at 60 °C for 72 h, sieved through a 2 mm mesh, and separated into coarse (>2 mm) and fine (<2 mm) fractions. Root biomass, coarse fragments, fine earth content, and gravimetric soil moisture were determined. Fine-earth subsamples were analysed for pH (H_2_O), organic matter, available P, available K, and total CaCO_3_ at the Laboratory Unit of the Department of Agricultural and Environmental Sciences, Polytechnic University of Santarém.

Total carbon and nitrogen were determined in both the bulk soil and the sand, silt, and clay fractions. Particle-size fractions were obtained by wet sieving using 0.250 mm and 0.053 mm sieves to separate sand, silt, and clay, respectively. Carbon and nitrogen concentrations were determined by dry combustion using a LECO TruSpec CHNS macro elemental analyser (LECO Corporation, St. Joseph, MI, USA) at the Instrumental Analysis Unit of the University of Santiago de Compostela (Spain).

Soil bulk density for the 0–10 cm layer was calculated as the mean of the values obtained for the 0–5 cm and 5–10 cm depth intervals.

### 2.5. Data Analysis

#### 2.5.1. Community Description

All analyses were performed in R version 4.4.1 [23]. The community was described using abundance (number of individuals captured), taxon richness (number of taxa), frequency of occurrence, and the relative abundance of taxa and functional groups per trap and sampling campaign. The dataset comprised 98 valid trap-visits. Phytophages and parasitoids were retained in the descriptive analyses but were not modelled because several stand–campaign combinations contained no captures, resulting in unstable estimates.

#### 2.5.2. Variation Among Stands and Sampling Campaigns

Variation in total abundance, taxon richness, and the abundance of predators, omnivores, decomposers, and taxa with unresolved or multiple trophic functions was analysed using generalized linear mixed models (GLMMs) fitted with the glmmTMB package 1.1.9 [24]. Stand, sampling campaign, and their interaction were included as fixed effects, and trap identity was included as a random intercept to account for repeated observations. For each response, Poisson, negative binomial type 1, negative binomial type 2, and Conway–Maxwell–Poisson distributions were compared, together with zero-inflated forms when appropriate. The distribution was selected using Akaike’s information criterion (AIC), considering only models with adequate convergence and a positive-definite Hessian matrix. The selected distributions were negative binomial type 2 for total abundance, decomposer abundance, and the abundance of taxa with unresolved or multiple trophic functions; Conway–Maxwell–Poisson for taxon richness and omnivore abundance; and negative binomial type 1 for predator abundance. Model adequacy was assessed using simulated residuals in DHARMa 0.4.6 [25], including tests of uniformity, dispersion, and zero inflation. Interactions were evaluated using likelihood-ratio tests and removed when not significant. Post hoc comparisons of estimated marginal means were performed with emmeans 1.10.2 [26] using Tukey adjustment.

#### 2.5.3. Soil Variable Selection and Principal Component Analysis

Twenty soil variables measured in the 0–10 cm layer were initially considered. Gravimetric moisture was excluded because it was not measured simultaneously in all stands, and total CaCO_3_ was excluded because all values were zero. Collinearity was evaluated using Pearson correlations and variance inflation factors (VIFs). VIFs were recalculated iteratively after removing the variable with the highest value until all retained variables had VIF < 3. The final set comprised fine material, silt, clay, roots, organic matter, pH, available K, the C/N ratio, and N in the clay fraction. These variables were standardized to zero mean and unit variance and subjected to principal component analysis (PCA) using the prcomp function in the stats package. The first two components were retained as orthogonal edaphic gradients. Their scores were subsequently standardized, producing PC1_z and PC2_z.

#### 2.5.4. Associations Between Edaphic Gradients and Arthropod Responses

The association between each PCA axis and stand identity was evaluated descriptively using separate linear models, with PCA scores as response variables and stand as the explanatory variable. Because PC1 was strongly associated with stand identity (R^2^ = 0.76), stand and the edaphic axes were not included simultaneously in subsequent models. Associations between the edaphic gradients and arthropod responses were evaluated using the 98 trap-visits. For each of the six responses described above, GLMMs included PC1_z, PC2_z, and sampling campaign as fixed effects and trap identity as a random intercept. Distribution selection and diagnostic assessment followed the same procedure described for the stand–campaign models. The 12 *p*-values associated with PC1 and PC2 across the six responses were corrected jointly using the Benjamini–Hochberg procedure [27]. Effects were expressed as rate ratios [exp(β)] with 95% confidence intervals.

#### 2.5.5. Taxonomic Composition and Edaphic Gradients

Associations between edaphic gradients and taxonomic composition were analysed using the 23 traps with complete sampling across all four campaigns. Taxon abundances were summed across campaigns within each trap and square-root transformed before calculating Bray–Curtis dissimilarities. Taxonomic composition was represented by two-dimensional non-metric multidimensional scaling (NMDS) using vegan 2.6.6.1 [28]. PC1 and PC2 were fitted to the ordination using envfit with 999 permutations. The two *p*-values obtained with envfit were adjusted jointly using the Benjamini–Hochberg procedure [27], and the marginal associations with composition were tested using PERMANOVA (adonis2; 999 permutations). Differences in multivariate dispersion among the three stands were assessed using betadisper and a permutation test with 999 permutations.

#### 2.5.6. Temporal Variation in Taxonomic Composition

Temporal variation in taxonomic composition was analysed from the 98 trap-visits. Taxon abundances were square-root transformed and Bray–Curtis dissimilarities were calculated. Two- and three-dimensional NMDS solutions were compared, and the three-dimensional solution was retained because it had lower stress. The overall effect of sampling campaign was tested using PERMANOVA with 999 permutations restricted within trap identity using the permute package 0.9.7 [29]. Differences in temporal trajectories among stands were assessed by adding the stand × campaign interaction; the main effect of stand was not tested because permutations were restricted within traps and there was only one stand of each forest type. Homogeneity of multivariate dispersion among campaigns was assessed using betadisper and a permutation test restricted within trap identity. Statistical significance was evaluated at α = 0.05.

## 3. Results

### 3.1. Community Structure

A total of 5511 arthropods were recorded from the 98 valid trap-visits obtained across 25 traps and four sampling campaigns; two samples were not recovered. The captures comprised 18 orders and 54 taxa, with a mean richness of 8.62 taxa per trap and campaign. Diptera, Acari, Hymenoptera, and Coleoptera accounted for most captures, although their relative contributions varied among stands and campaigns (Figure 2).

Taxa with unresolved or multiple trophic functions were the most abundant functional group (3005 individuals; 54.5% of all captures), mainly comprising Diptera (49.0% of this group), Acari (36.6%), and Opiliones (7.0%). Predators comprised 938 individuals (17.0%) and were dominated by other Staphylinidae (43.6%), Araneae (26.2%), and Scydmaeninae (16.3%). Omnivores comprised 913 individuals (16.6%) and were almost entirely represented by Formicidae (98.7%). Phytophages accounted for 375 individuals (6.8%) and were dominated by Scolytinae (60.5%), parasitoids comprised 152 individuals (2.8%), and decomposers were the least abundant group (128 individuals; 2.3%), mainly represented by Diplopoda (14.8%), Psocoptera (13.3%), Aphodiidae (12.5%), and Isopoda (12.5%) (Figure 3).

Omnivores, phytophages, and parasitoids were absent from 44.9%, 48.0%, and 44.9% of trap-visits, respectively. The most conspicuous descriptive pattern was the high abundance of phytophages in the pine stand in April (39.8 individuals per trap), largely driven by Scolytinae (Figure S1).

### 3.2. Variation Among Stands and Sampling Campaigns

The stand × campaign interaction was significant for taxon richness (χ^2^ = 27.96, df = 6, *p* < 0.001), predator abundance (χ^2^ = 29.17, df = 6, *p* < 0.001), and the abundance of taxa with unresolved or multiple trophic functions (χ^2^ = 14.27, df = 6, *p* = 0.0268). Taxon richness did not differ among stands in July or April. In October, richness was higher in the holm oak stand than in the pine and Pyrenean oak stands, whereas in February it was higher in the pine than in the Pyrenean oak stand, with the holm oak stand occupying an intermediate position. Predator abundance did not differ among stands in July or April but was lower in the Pyrenean oak stand than in the pine and holm oak stands in October and February. Taxa with unresolved or multiple trophic functions did not differ among stands in July, February, or April; in October, their abundance was lower in the Pyrenean oak than in the holm oak stand, while the pine stand was intermediate (Figure 4).

For total abundance, the interaction was slightly above the significance threshold (χ^2^ = 12.41, df = 6, *p* = 0.053), and the final model therefore retained additive effects. Total abundance differed among stands (χ^2^ = 21.94, df = 2, *p* < 0.001) and campaigns (χ^2^ = 96.29, df = 3, *p* < 0.001); it was lower in the Pyrenean oak stand than in the pine and holm oak stands, highest in April, and lowest in February. The stand × campaign interactions were also non-significant for omnivores (χ^2^ = 11.63, df = 6, *p* = 0.071) and decomposers (χ^2^ = 10.11, df = 6, *p* = 0.120). Omnivore abundance differed among stands (χ^2^ = 38.56, df = 2, *p* < 0.001) and campaigns (χ^2^ = 85.33, df = 3, *p* < 0.001), being lower in the Pyrenean oak stand and highest in July. Decomposer abundance varied among campaigns (χ^2^ = 13.34, df = 3, *p* = 0.004) but not among stands (χ^2^ = 3.77, df = 2, *p* = 0.152), with higher estimates in October and April (Figure 4). All retained models converged adequately and showed no significant deviations in residual uniformity, dispersion, or zero inflation.

Temporal variation within functional groups was mainly associated with changes in their dominant taxa (Figure S2). Within the group with unresolved or multiple trophic functions, Diptera and Acari declined in February and increased again in April in all three stands. The spring increase was particularly marked for Diptera in the pine stand, while both Diptera and Acari contributed to the increase observed in the two oak stands. Opiliones showed a different pattern, with a pronounced peak in the holm oak stand in October and smaller increases in the pine and holm oak stands in April. Predator dynamics reflected changes in different taxa. The high predator abundance in the holm oak stand in October was largely associated with Scydmaeninae, whereas the April increase in the pine and holm oak stands was mainly driven by other Staphylinidae. In February, Araneae contributed strongly to predator abundance in the pine stand and Carabidae in the holm oak stand. Omnivore patterns almost entirely reflected changes in Formicidae, which were abundant in July in the pine and holm oak stands, nearly absent during October and February, and increased again in April. No single taxon consistently accounted for the seasonal variation in decomposers, whose abundance reflected smaller changes in several taxa.

### 3.3. Edaphic Gradients

After VIF-based selection, the nine retained soil variables had VIF values below 2. PC1 explained 30.3% of the edaphic variation and had its largest positive loadings for C/N ratio (0.493), silt (0.479), fine material (0.388), clay (0.322), and pH (0.276), together with a negative loading for available K (−0.416). PC2 explained 18.7% and was mainly defined by positive loadings of N in the clay fraction (0.631), organic matter (0.552), and roots (0.464). Together, the two axes explained 49.0% of the edaphic variation (Figure 5). PC1 was strongly associated with stand identity (R^2^ = 0.762), whereas this association was moderate for PC2 (R^2^ = 0.373). Mean PC1 scores were highest in the maritime pine stand (2.45 ± 0.74), intermediate in the holm oak stand (0.15 ± 0.92), and lowest in the Pyrenean oak stand (−1.38 ± 0.83). Thus, the pine stand was positioned towards finer-textured soils with higher pH and C:N ratio and lower available K, whereas the Pyrenean oak stand occurred towards the opposite end of this gradient. Mean PC2 scores were negative in the holm oak stand (−0.96 ± 0.52) and positive in the Pyrenean oak (0.52 ± 1.27) and pine stands (0.88 ± 1.39), although their ranges overlapped.

Pearson correlations among the candidate soil variables are provided in Figure S3.

### 3.4. Arthropod Responses to Edaphic Gradients

Total arthropod abundance increased by 21.4% for each one-standard-deviation increase in PC1 (rate ratio [RR] = 1.214, 95% CI = 1.074–1.372, adjusted *p* = 0.0077). Omnivore abundance showed the strongest response: it increased by 136.7% along PC1 (RR = 2.367, 95% CI = 1.588–3.529, adjusted *p* < 0.001) and decreased by 54.8% along PC2 (RR = 0.452, 95% CI = 0.300–0.679, adjusted *p* < 0.001). No significant associations with either axis were detected for taxon richness, predators, decomposers, or taxa with unresolved or multiple trophic functions (Figure 6). All edaphic GLMMs converged and had adequate diagnostics.

### 3.5. Taxonomic Composition and Edaphic Gradients

The annual two-dimensional NMDS had a stress value of 0.141. Both edaphic axes were significantly associated with the ordination: PC1 had R^2^ = 0.559 (adjusted *p* = 0.004) and PC2 had R^2^ = 0.277 (adjusted *p* = 0.041). In marginal PERMANOVA tests, PC1 accounted for 21.5% of the variation in taxonomic composition (F = 6.45, *p* = 0.001), whereas PC2 accounted for 12.8% (F = 3.82, *p* = 0.003). The model including both edaphic axes explained 33.2% of the total variation. Mean distances to group centroids were 0.183 in the pine, 0.204 in the holm oak, and 0.206 in the Pyrenean oak stand, with no significant differences in multivariate dispersion among stands (F_2,20_ = 0.79, *p* = 0.502; Figure 7).

### 3.6. Temporal Variation in Taxonomic Composition

The temporal NMDS had stress values of 0.221 in two dimensions and 0.159 in three dimensions; consequently, the three-dimensional solution was retained and is presented through the NMDS1–NMDS2 and NMDS1–NMDS3 projections (Figure 8). Sampling campaign explained 28.7% of the variation in taxonomic composition (F = 12.59, *p* = 0.001). The stand × campaign interaction explained 21.4% (F = 4.61, *p* = 0.001), indicating that temporal community trajectories differed among the three stands.

Multivariate dispersion also differed among campaigns (F = 4.17, *p* = 0.015). October had the greatest mean distance to the campaign centroid (0.368), indicating the highest within-campaign heterogeneity, whereas April had the lowest (0.305); February (0.354) and July (0.315) were intermediate. Thus, the PERMANOVA result may reflect both shifts in mean community composition and differences in within-campaign heterogeneity.

## 4. Discussion

Ground-dwelling arthropod communities showed pronounced temporal variation and marked differences among the three forest stands. Total abundance, taxon richness, and several functional groups varied among stands, sampling campaigns, or through their interaction. Taxonomic composition also changed seasonally, and these changes differed among stands. Edaphic gradients were associated with both community abundance and composition.

### 4.1. Taxonomic and Functional Community Structure

Most organisms were identified at family or order level. Although this higher-taxon approach involves some information loss, it can reveal broad seasonal patterns in epigeic arthropod communities [30]. Because many soil-associated arthropods use different resources, the functional groups used here should be interpreted as the main or potential functions of each taxon rather than as exclusive feeding categories [2].

The group with unresolved or multiple trophic functions was dominated by Diptera, Acari, and Opiliones. Diptera and Acari encompass consumers with diverse feeding habits and trophic positions [2,31], while harvestmen may consume live prey, carrion, and occasionally plant or fungal material [2]. Identification at broad taxonomic levels prevented assignment to a single predominant function and may have obscured taxon-specific responses, as the taxa included in this category may respond differently—or even in opposite directions—to seasonal and edaphic variation. Moreover, the group-level pattern was strongly influenced by the numerically dominant Diptera and Acari. Its response should therefore not be assumed to represent a shared response among all constituent taxa, and finer taxonomic identification could alter the estimated functional structure.

Predators were well represented, particularly by Araneae, Scydmaeninae, and other Staphylinidae. Scydmaeninae commonly prey on small litter- and soil-dwelling arthropods [32]. Their occurrence is consistent with the importance of prey availability, litter structure, refuges, and surface microclimate for epigeic predators [33,34,35,36]. Omnivores were almost exclusively Formicidae, which were included in this group because Iberian ants include mostly generalist omnivores. However, some species have more specialized diets, and species-level identification could add detail to their functional interpretation [37].

Phytophagous arthropods and parasitoids were scarce, as expected from a method that primarily samples surface-active organisms [38]. The April increase in phytophagous arthropods in the pine stand was driven mainly by Scolytinae, a group that includes important pests of natural and planted pine forests [39]. This spring peak may reflect increased bark beetle activity as temperatures rise [40]. A similar spring flight period has been reported for the Scolytinae *Ips sexdentatus* (Börner, 1776) in *P. pinaster* forests [41,42].

Decomposers were comparatively scarce. This may partly reflect the sampling method, as pitfall traps primarily capture arthropods active on the soil surface and may underrepresent organisms living within litter or soil [38]. In contrast, Deng et al. [11], who used direct extraction from litter and upper-soil samples, found that saprophagous arthropods represented a substantial proportion of the sampled community. The different sampling methods may partly explain this contrast. Also, Collembola were not included in the present study, and some taxa potentially involved in decomposition remained in the group with unresolved or multiple trophic functions, which may have reduced the estimated abundance and altered the inferred composition of the decomposer group.

### 4.2. Temporal Variation Within and Among Forest Stands

Total abundance was lowest in February and highest in April, consistent with seasonal activity under a Mediterranean–sub-Atlantic transitional climate. Rising spring temperatures and resource availability may stimulate arthropod activity, whereas low winter temperatures restrict it. Greater spring than autumn abundance and richness have also been reported for epigeic arthropods in northwestern Portugal [30], and soil temperature and moisture are recognized determinants of arthropod communities [10,43]. Total abundance was lower in the Pyrenean oak stand than in the pine and holm oak stands, potentially reflecting differences in vegetation, litter, and resource conditions [11,44].

Taxon richness was generally high in April, while the holm oak stand also showed high richness in October. Spring and autumn diversity peaks occur in other Mediterranean insect assemblages [45], and some insects associated with oaks remain active or become more abundant in autumn [46].

Taxa with unresolved or multiple trophic functions reached their lowest abundance in February and increased again in April, contributing strongly to the overall abundance pattern. Diptera and Acari followed this general pattern in all three stands, although the spring increase was particularly marked for Diptera in the pine stand, whereas both taxa contributed to the increase in the two oak stands. Opiliones differed from this pattern, with a pronounced peak in the holm oak stand in October and smaller increases in the pine and holm oak stands in April. Because Diptera and Acari encompass species with widely different ecological requirements, the variation within this functional group probably reflects several ecological processes rather than a single mechanism [2,31].

Predator abundance also varied differently among stands, being lower in the Pyrenean oak stand than in the other stands in October and February, but not in July or April. In October, the high abundance in the holm oak stand was mainly associated with Scydmaeninae. In February, Araneae contributed strongly in the pine stand, whereas Carabidae made the largest contribution in the holm oak stand. By April, other Staphylinidae accounted for most of the increase in the pine and holm oak stands. This taxonomic turnover may reflect differences in phenology, prey use, litter conditions, and microhabitat requirements [35,36,47].

Omnivores, represented almost entirely by Formicidae, were less abundant in the Pyrenean oak stand and reached their highest abundance in July. Formicidae were abundant in July in the pine and holm oak stands, nearly absent in October and February, and increased again in April. This pattern is consistent with the strong influence of temperature on ant metabolism and foraging, although responses vary among species and habitats [48,49].

Decomposers varied among campaigns but not among stands, with higher overall abundance in October and April, in agreement with the seasonality of Mediterranean litter and soil macroinvertebrate assemblages [50]. Their temporal variation was not consistently associated with a single dominant taxon. Psocoptera contributed to the values observed in July, Aphodiidae were particularly relevant in the holm oak stand in October, and Diplopoda made a greater contribution in February. In April, the taxa contributing to decomposer abundance differed among stands.

Seasonal variation was also evident in taxonomic composition. Sampling campaign accounted for 28.7% of compositional variation, indicating that seasonal change affected not only abundance and richness but also the identity and relative abundance of the taxa recorded. The significant interaction between campaign and stand further showed that compositional changes did not occur in the same way in all three stands. Such differences may arise because seasonal changes in litter, microclimate, soil conditions, and vegetation do not affect all forest arthropod assemblages equally [11,43,44].

Variation among traps was greatest in October and lowest in April, indicating that autumn samples differed more strongly from one another than spring samples. This greater autumn heterogeneity may reflect differences among taxa in the timing of their responses to changing environmental conditions. Because multivariate dispersion also differed among campaigns, the PERMANOVA result cannot be attributed exclusively to shifts in average community composition. Nevertheless, the three-dimensional ordination showed seasonal changes in composition whose direction differed among stands.

### 4.3. Relationships with Edaphic Gradients

The first two PCA axes summarized approximately half of the observed edaphic variation. PC1 was associated with greater proportions of silt, clay, and fine material, higher C:N ratio and pH, and lower available potassium, whereas PC2 was associated mainly with clay-bound nitrogen, organic matter, and root abundance. These integrated gradients were related to arthropod community organization, consistent with reported associations of soil texture, moisture, temperature, pH, organic matter, and C:N ratio with soil arthropods [10,11].

Total arthropod abundance increased along PC1, indicating greater overall activity-density towards the positive end of this gradient. The greater proportion of fine particles may contribute to water retention and more stable near-surface microclimatic conditions [13], potentially favouring arthropod activity.

Omnivores showed the strongest associations with the edaphic gradients, increasing along PC1 and decreasing along PC2. Because this group was almost entirely composed of Formicidae, these patterns primarily reflect ant activity. Ant communities may respond to both soil properties and vegetation structure [51,52]. Therefore, the decline in omnivore abundance along PC2 indicates a negative association with the combined gradient of organic matter, root abundance, and clay-bound nitrogen.

PC1 and PC2 were also associated with taxonomic composition and jointly accounted for 33.2% of its variation. Thus, changes along the edaphic gradients were accompanied by differences in the identity and relative abundance of the taxa recorded. Scolytinae and Curculionidae were positioned towards increasing PC1 scores, that is, towards finer-textured soils with higher pH and C:N ratio and lower available potassium, whereas Isopoda and Hemiptera occurred towards the opposite end of this gradient. Along PC2, Isopoda and Chilopoda were positioned towards higher organic matter, root abundance, and clay-bound nitrogen, while Scydmaeninae, Opiliones, and Formicidae were positioned in the opposite direction.

Finally, PC1 was strongly associated with stand identity. Its associations with arthropod abundance and composition should therefore be interpreted with caution because soil conditions cannot be fully separated from other differences among plots.

### 4.4. Limitations and Ecological Implications

Only one stand of each forest type was studied. The traps characterized spatial variation within each stand but did not provide independent replication at the forest-type level. Consequently, the observed differences among stands cannot be attributed exclusively to the dominant tree species. Other stand-specific characteristics, including canopy structure, litter depth and composition, deadwood availability, floral resources, proximity to water, management history, and local microclimatic conditions, may also have contributed to the observed patterns. Although some habitat characteristics were described using the available information, they were not all quantified systematically during arthropod sampling.

Soil properties were measured once, which is suitable for relatively stable attributes such as texture but does not represent seasonal variation in moisture and temperature. Pitfall captures depend on both the number of organisms present and their movement across the soil surface. Therefore, they do not measure absolute abundance and capture fewer organisms that live in vegetation or within the soil, or that have limited mobility [38]. This limitation is particularly relevant for Diptera and other predominantly flying taxa, whose activity is less consistently represented by pitfall catches. Identification mainly at family or order level, together with the exclusion of Collembola, may also have biased the taxonomic and functional interpretation of the community.

Finally, the four campaigns covered only one annual cycle. Long-term studies and repeated measurements of soil and litter microclimate are needed to assess the stability and mechanisms of the observed responses. Even with these limitations, the combined analysis of abundance, richness, functional structure, and taxonomic composition showed that the communities changed through time and were associated with edaphic gradients. A mosaic of forest conditions may support complementary taxa and functions, but this remains a hypothesis because forest types were not independently replicated.

## 5. Conclusions

Ground-dwelling arthropod communities in holm oak, Pyrenean oak, and maritime pine stands showed pronounced seasonal changes. The taxa contributing to functional groups also varied among campaigns and stands: for example, Scydmaeninae contributed strongly to predator abundance in the holm oak stand in October, Araneae and Carabidae made important winter contributions in the pine and holm oak stands, respectively, and other Staphylinidae drove the spring increase in predators. Overall, temporal changes differed among stands for taxon richness, predators, and taxa with unresolved or multiple trophic functions, whereas total abundance, omnivores, and decomposers followed more similar seasonal patterns across stands. Changes in functional-group abundance were often accompanied by changes in the taxa contributing to each group.

Total abundance and Formicidae-dominated omnivore abundance increased along PC1, indicating an association between greater arthropod activity and finer-textured soils with higher pH and C:N ratio and lower available potassium. Omnivores decreased along PC2, which mainly represented increasing organic matter, root abundance, and clay-bound nitrogen. Both axes were associated with taxonomic composition and jointly accounted for 33.2% of its variation. Although soil and stand effects cannot be separated completely, the results show that integrated edaphic gradients were associated with arthropod abundance and taxonomic composition.

Formicidae, epigeic predators, and Scolytinae emerged as particularly informative groups for further research. Formicidae showed strong seasonal and edaphic associations, predator taxa displayed marked turnover among stands and campaigns, and Scolytinae accounted for the pronounced spring increase in phytophagous arthropods in the pine stand. Finer taxonomic identification of Diptera and Acari would also help clarify the ecological processes represented by the abundant group with unresolved or multiple trophic functions. The study therefore provides a baseline and identifies focal groups for long-term research in forests of the Mediterranean–sub-Atlantic transition.

## Figures and Tables

**Figure 1:**
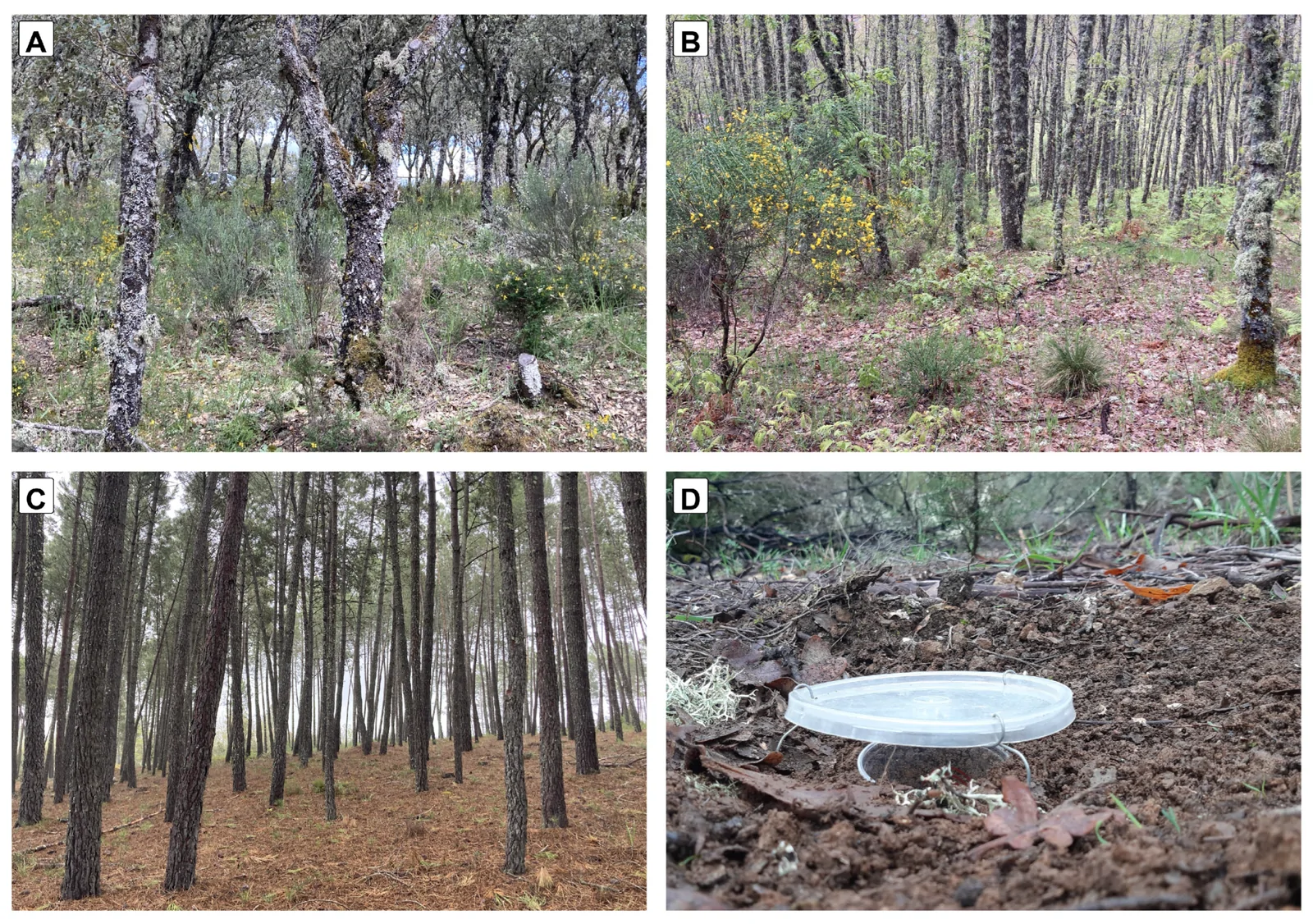
Representative views of the forest stands and sampling method used in this study: (**A**) holm oak (*Quercus rotundifolia*) woodland; (**B**) Pyrenean oak (*Quercus pyrenaica*) woodland; (**C**) maritime pine (*Pinus pinaster*) plantation; and (**D**) pitfall trap installed at ground level and protected by a raised cover.

**Figure 2:**
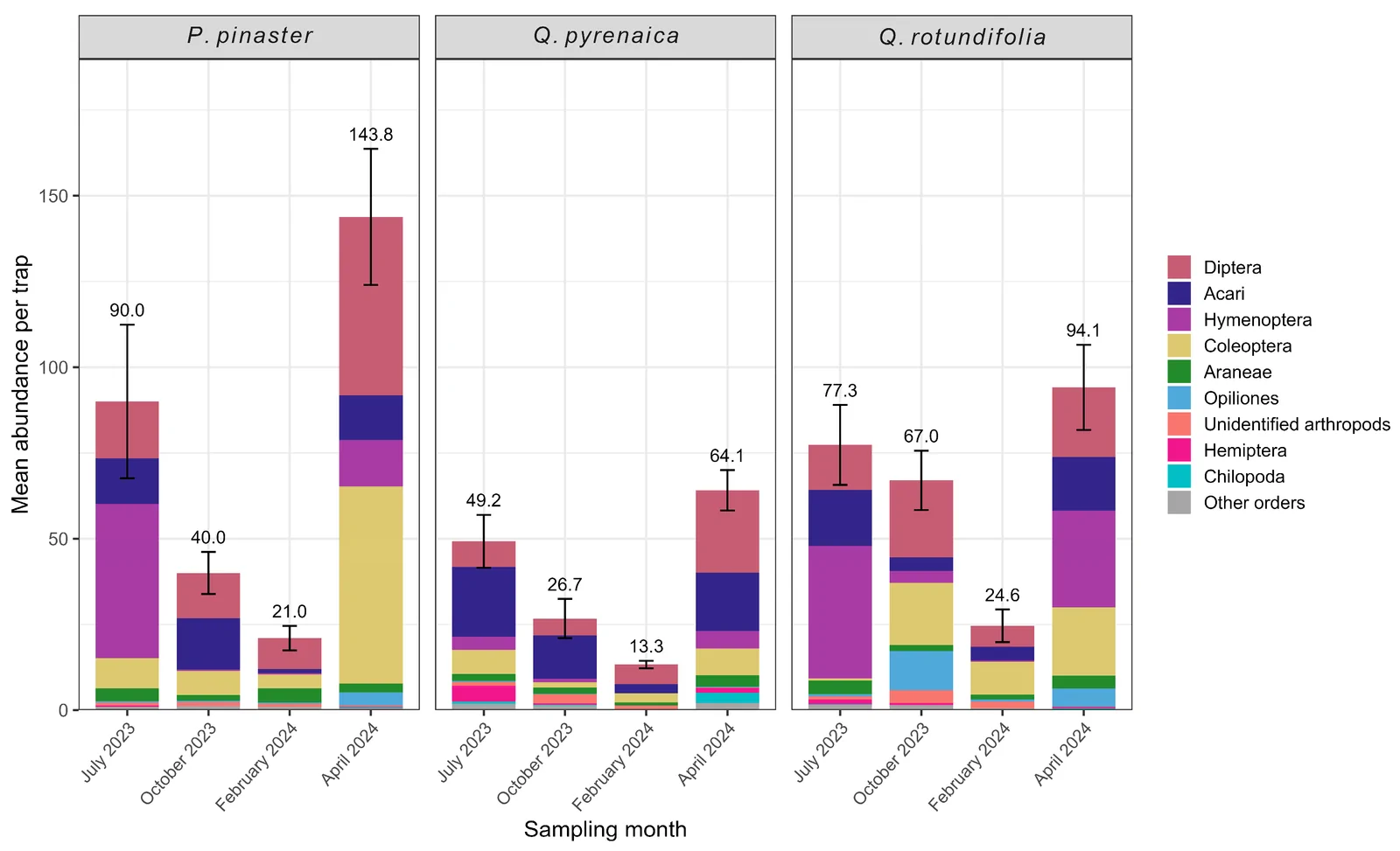
Taxonomic composition and mean arthropod abundance per trap in the three forest stands and four sampling campaigns. Error bars represent the standard error of total abundance.

**Figure 3:**
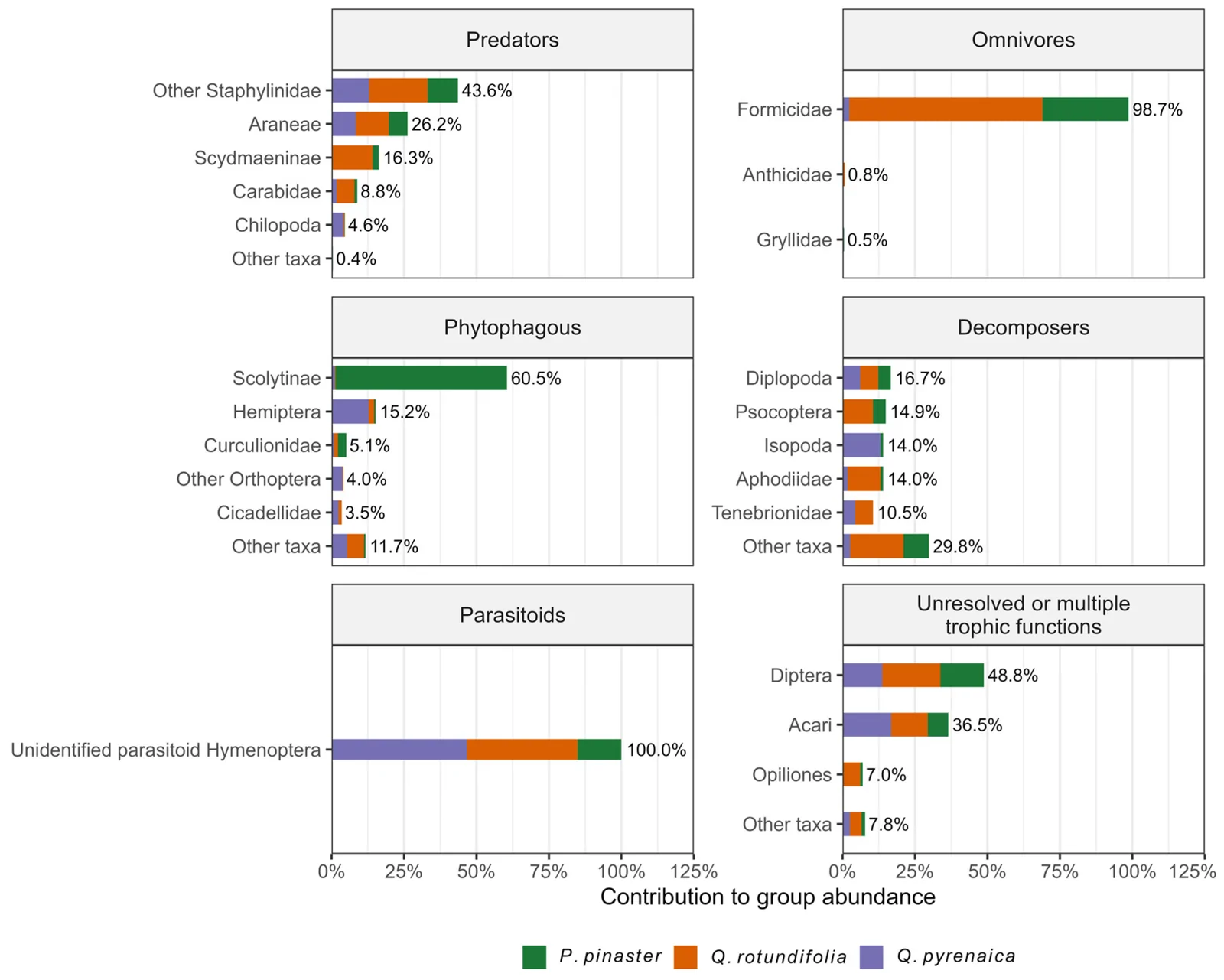
Contribution of the most abundant taxa to each functional group, partitioned among the three forest stands. Percentages indicate each taxon’s contribution to the total abundance of its functional group across all stands.

**Figure 4:**
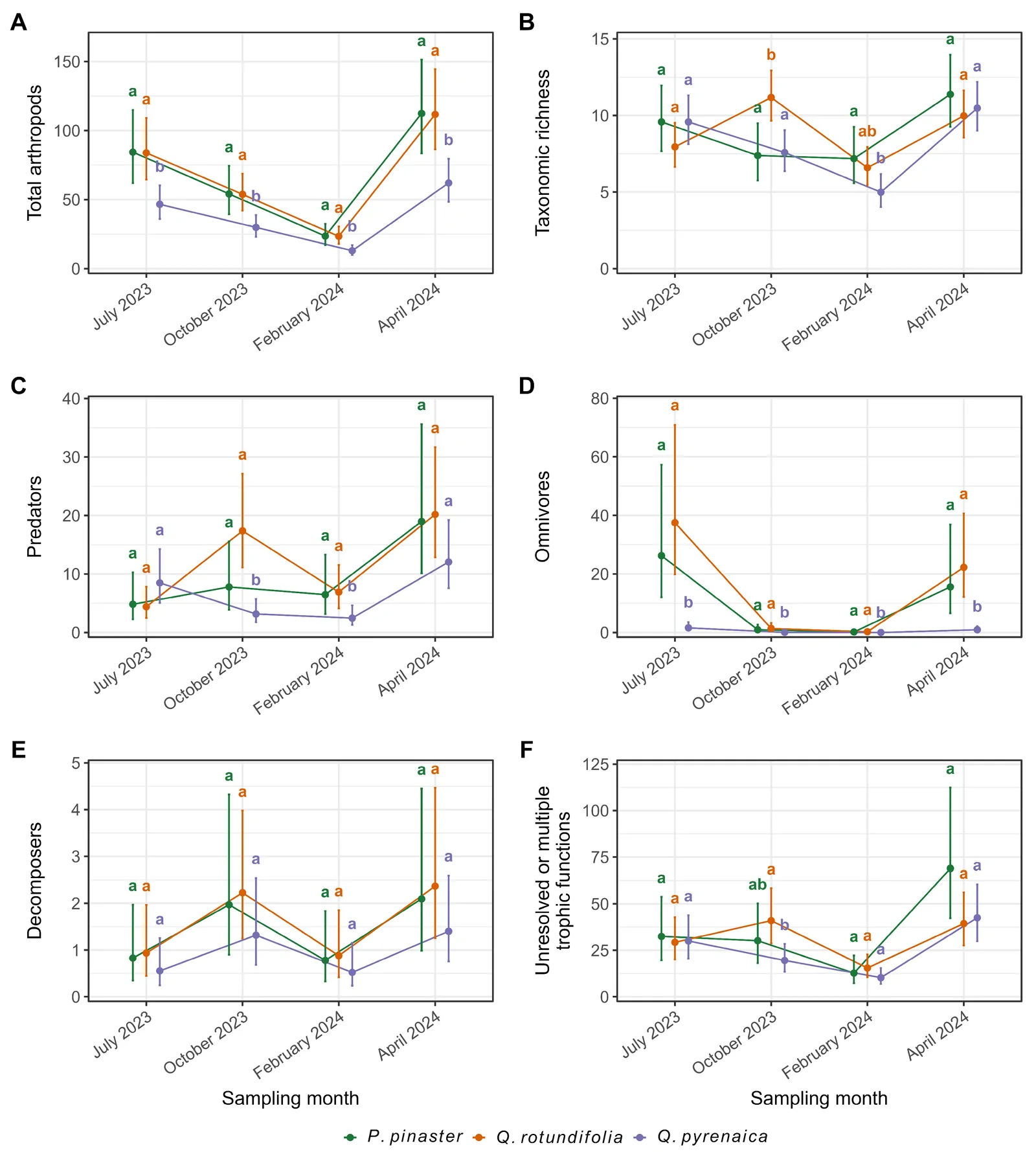
Estimated marginal means (points) and 95% confidence intervals for (**A**) total abundance, (**B**) taxon richness, and the abundance of (**C**) predators, (**D**) omnivores, (**E**) decomposers, and (**F**) taxa with unresolved or multiple trophic functions per trap in the three forest stands and four sampling campaigns. Within each campaign, different letters indicate significant differences among stands in Tukey-adjusted comparisons (*p* < 0.05).

**Figure 5:**
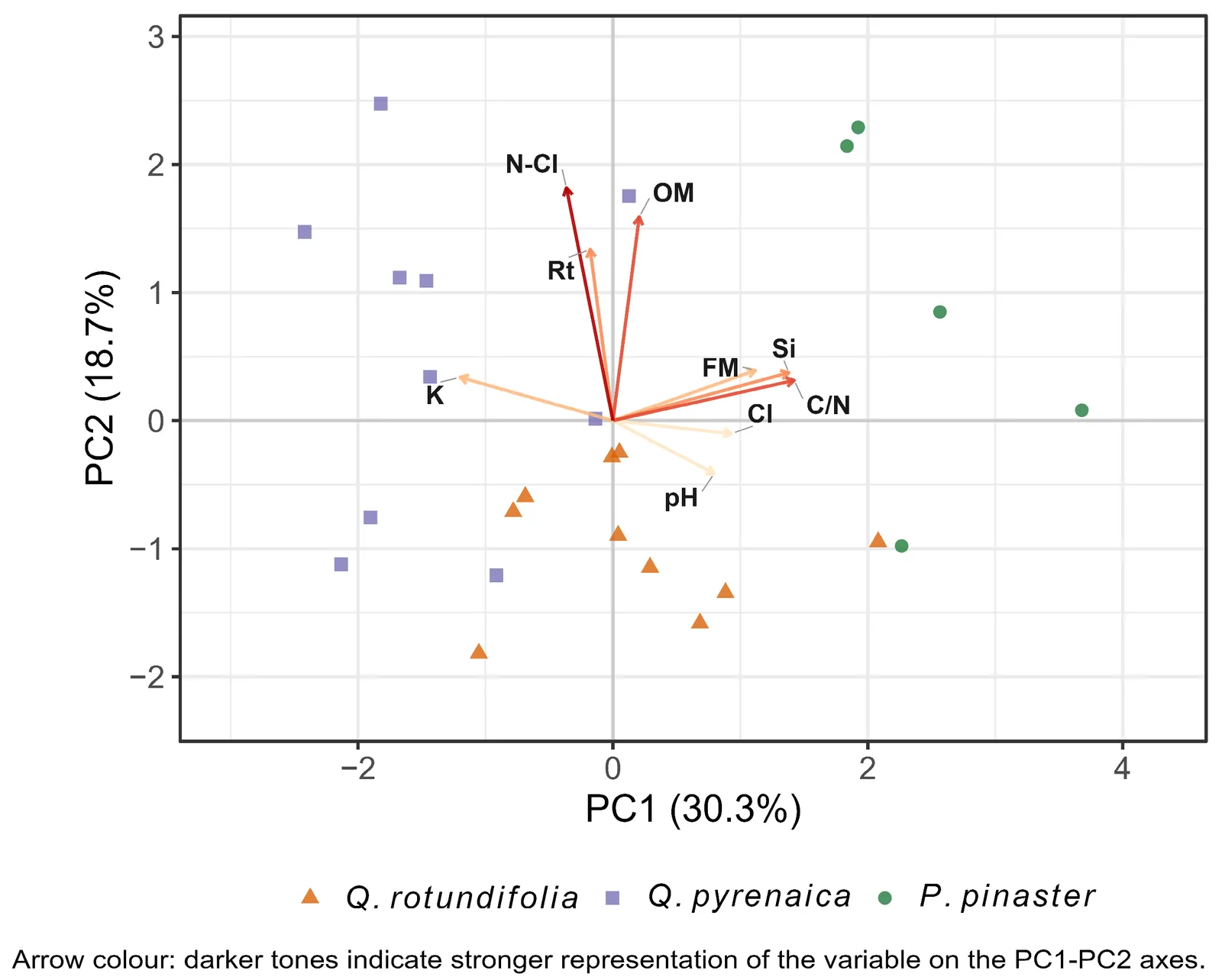
Principal component analysis of soil properties. Points represent traps and arrows show the direction of increasing values for the nine variables retained after VIF selection; arrow length and color indicate their relative contribution to the displayed axes. PC1 and PC2 explained 30.3% and 18.7% of total edaphic variation, respectively. FM, fine material; Si, silt; Cl, clay; Rt, roots; OM, organic matter; pH, soil pH; K, available potassium; C/N, carbon-to-nitrogen ratio; N-Cl, nitrogen in the clay fraction.

**Figure 6:**
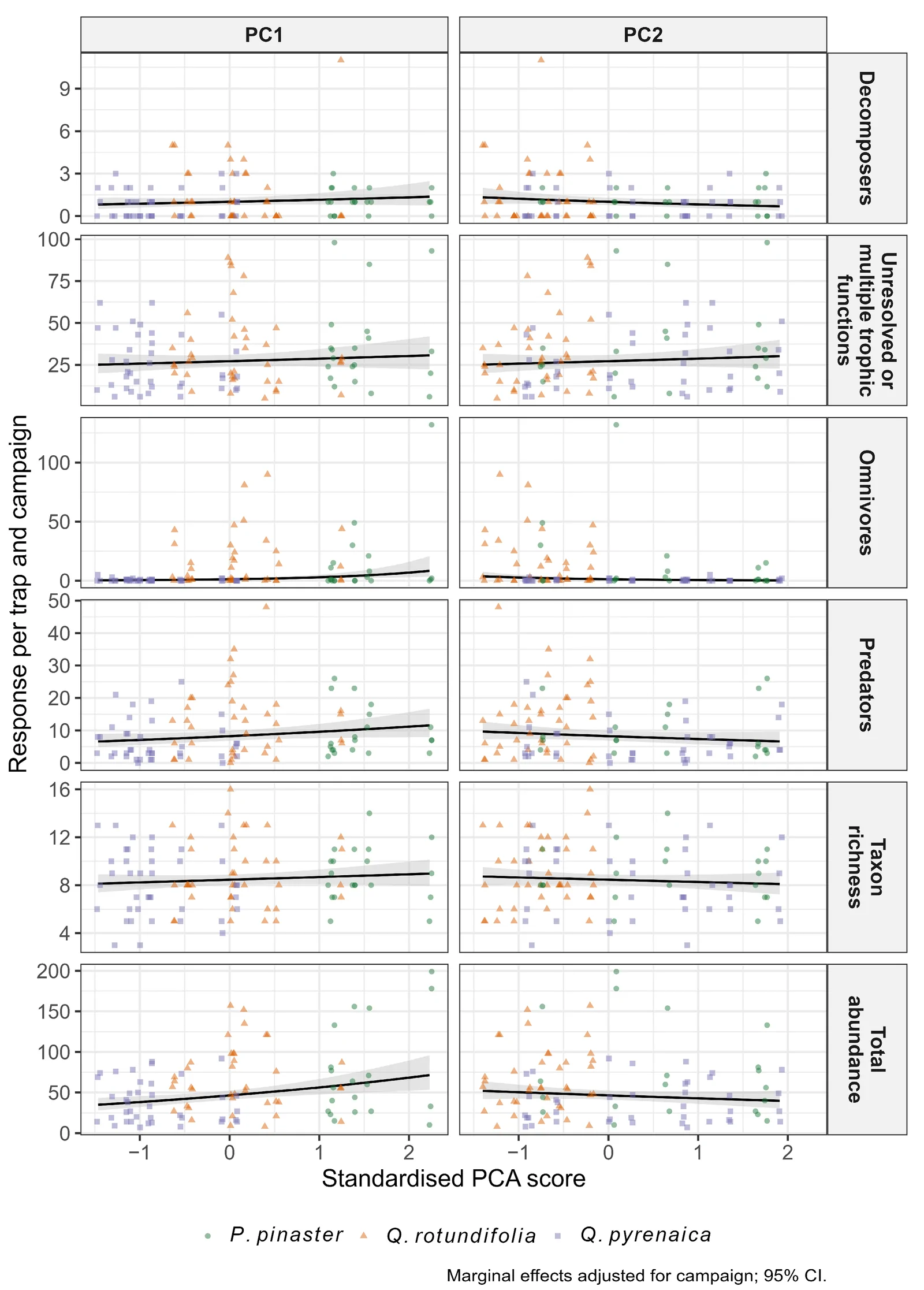
Estimated relationships between standardized PC1 or PC2 scores and expected arthropod responses after accounting for sampling campaign and repeated observations from the same trap. Points represent observed values; colors and symbols identify forest stands. Lines show GLMM predictions and shaded bands represent 95% confidence intervals. PC1 and PC2 scores were standardized to a mean of zero and a standard deviation of one.

**Figure 7:**
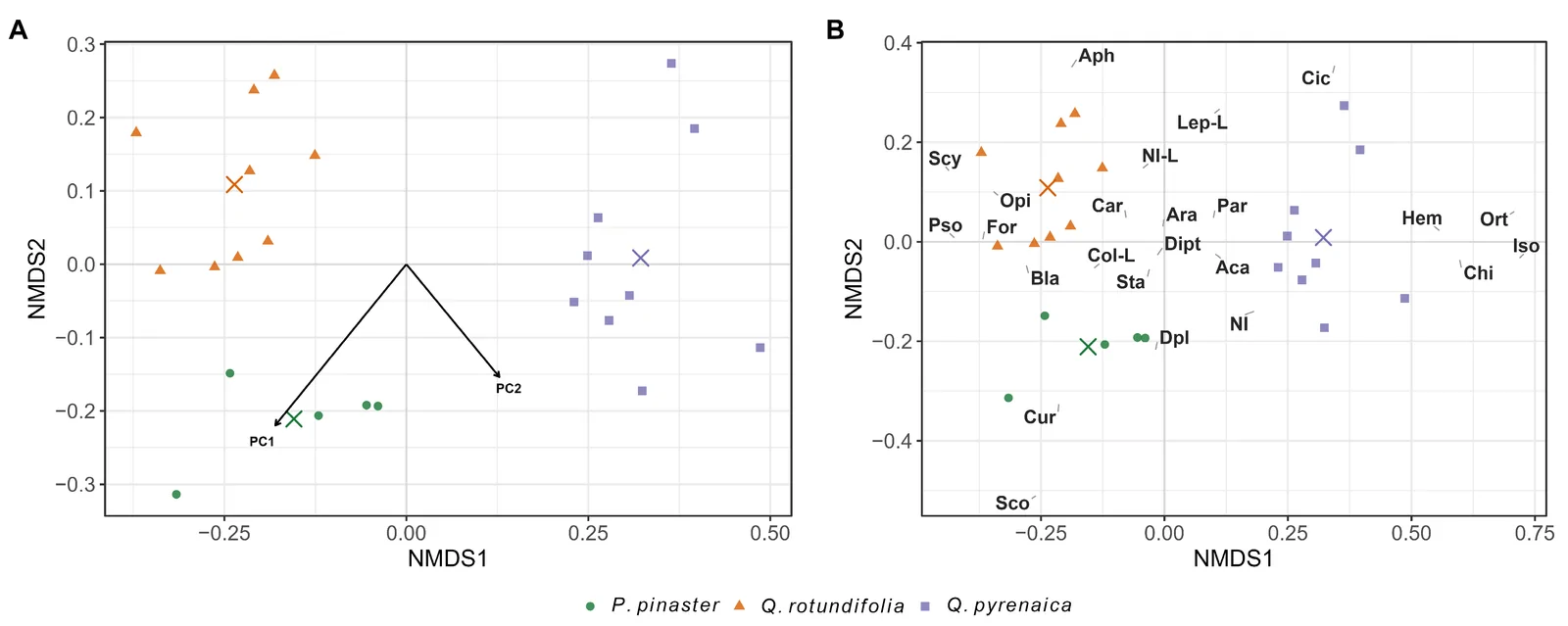
Arthropod taxonomic composition and edaphic gradients based on NMDS of taxon abundances accumulated within each trap. Colors and symbols identify forest stands, and large × symbolsrepresent their centroids. (**A**) Association of PC1 and PC2 with the ordination. (**B**) Positions of the 24 taxa occurring in at least three traps and having a total abundance of at least 10 individuals. Aca, Acari; Aph, Aphodiidae; Ara, Araneae; Bla, Blattodea; Car, Carabidae; Chi, Chilopoda; Cic, Cicadellidae; Col-L, Coleoptera larvae; Cur, Curculionidae; Dipt, Diptera; Dpl, Diplopoda; For, Formicidae; Hem, Hemiptera; Iso, Isopoda; Lep-L, Lepidoptera larvae; NI, unidentified organisms; NI-L, unidentified larvae; Opi, Opiliones; Ort, other Orthoptera; Par, unidentified parasitoid Hymenoptera; Pso, Psocoptera; Sco, Scolytinae; Scy, Scydmaeninae; Sta, Other Staphylinidae.

**Figure 8:**
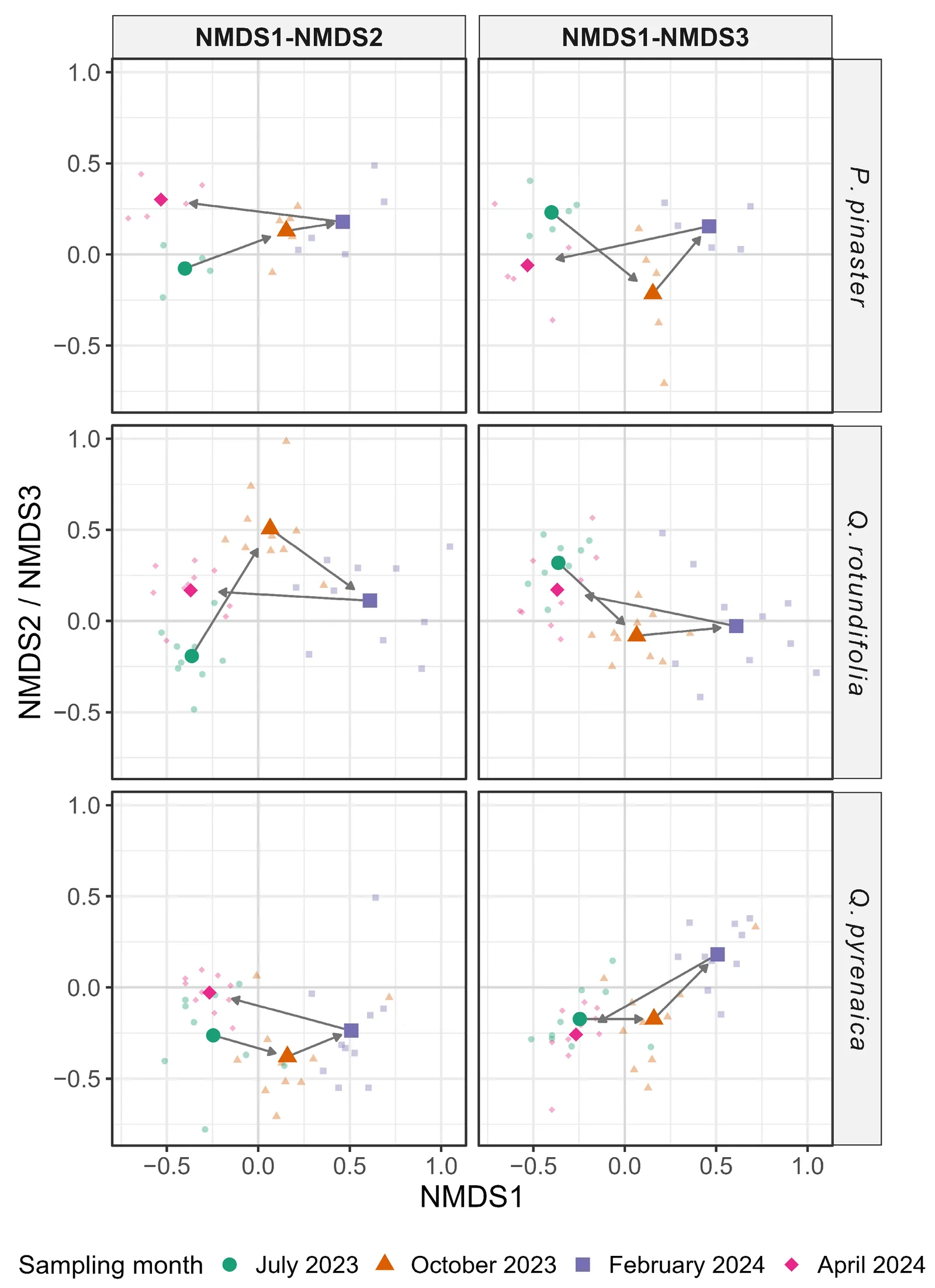
Temporal variation in taxonomic composition shown by the NMDS1–NMDS2 and NMDS1–NMDS3 projections of the three-dimensional solution. Each point represents the community observed in one trap during one campaign. Centroids indicate the mean position of each stand in each campaign, and arrowed segments connect consecutive campaigns to show the direction of temporal trajectories.

## Data Availability

The dataset generated during the current study is available in the Zenodo repository under a Creative Commons Attribution 4.0 International License (CC-BY 4.0): https://doi.org/10.5281/zenodo.21932520.

## Supplementary Materials

The following supporting information can be downloaded at https://www.mdpi.com/article/10.3390/insects17100988/s1. Figure S1: Boxplot of the temporal variation in phytophagous and parasitoid abundance across forest stands. Points represent individual traps. The horizontal line within each box indicates the median, the box represents the interquartile range, and the whiskers extend to values within 1.5 times the interquartile range. Figure S2: Temporal variation in the main taxa assigned to each functional group. Points represent mean abundance per trap and error bars represent standard errors of the mean. Figure S3: Pearson correlations among soil properties measured at 0–10 cm depth. The lower triangle shows correlations as circles, with circle size proportional to the absolute correlation coefficient (|r|), whereas the upper triangle displays the corresponding Pearson correlation coefficients. Red indicates positive correlations and blue indicates negative correlations; stronger colour intensity represents stronger associations. Rt, roots; CM, coarse material; FM, fine material; OM, organic matter; Sa, sand; Si, silt; Cl, clay; BD, bulk density; P, available phosphorus; K, available potassium; TC, total carbon; TN, total nitrogen; C/N, carbon-to-nitrogen ratio; C-Sa, carbon in the sand fraction; C-Si, carbon in the silt fraction; C-Cl, carbon in the clay fraction; N-Sa, nitrogen in the sand fraction; N-Si, nitrogen in the silt fraction; N-Cl, nitrogen in the clay fraction. Table S1: Abundance of every recorded taxon by forest stand and sampling campaign. Table S2: Taxonomic categories recorded in pitfall traps and their assignment to functional groups.
