# Supplementary material for: Soil–Arthropod Interactions Across Forest Types in a Mediterranean Mountain Landscape
Source: Insects. 2026 Sep 24;17(10):988. doi: 10.3390/insects17100988 (PMC13705440; doi:10.3390/insects17100988)
Supplement: Supplementary file 1 [file insects-17-00988-s001.zip › Supplementary material.pdf]

Supplementary material

Soil–arthropod interactions across forest types in a Mediterranean mountain landscape

María Villa [1,*], Diogo Angélico [1], Marina Castro [1]

[1] CIMO, LA SusTEC, Instituto Politécnico de Bragança, Campus de Santa Apolónia, 5300-253 Bragança, Portugal; marina.castro@unipb.pt; mariavilla@unipb.pt

*mariavilla@unipb.pt; Tel.: +351 273 303 344

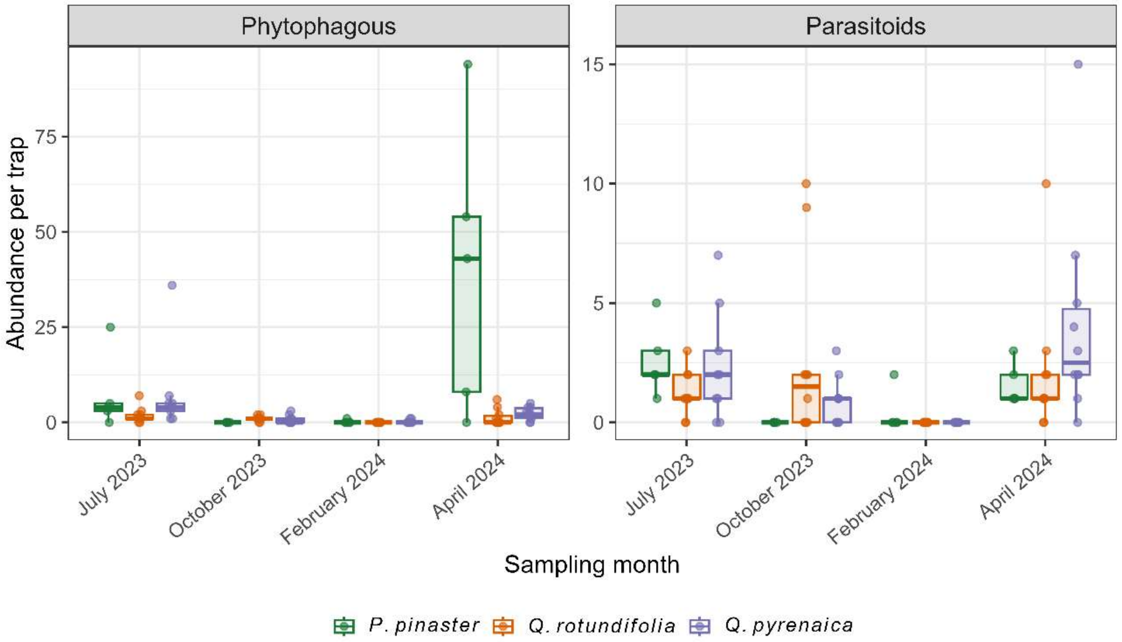

Figure S1. Boxplot of the temporal variation in phytophagous and parasitoid abundance across forest stands. Points represent individual traps. The horizontal line within each box indicates the median, the box represents the interquartile range, and the whiskers extend to values within 1.5 times the interquartile range.

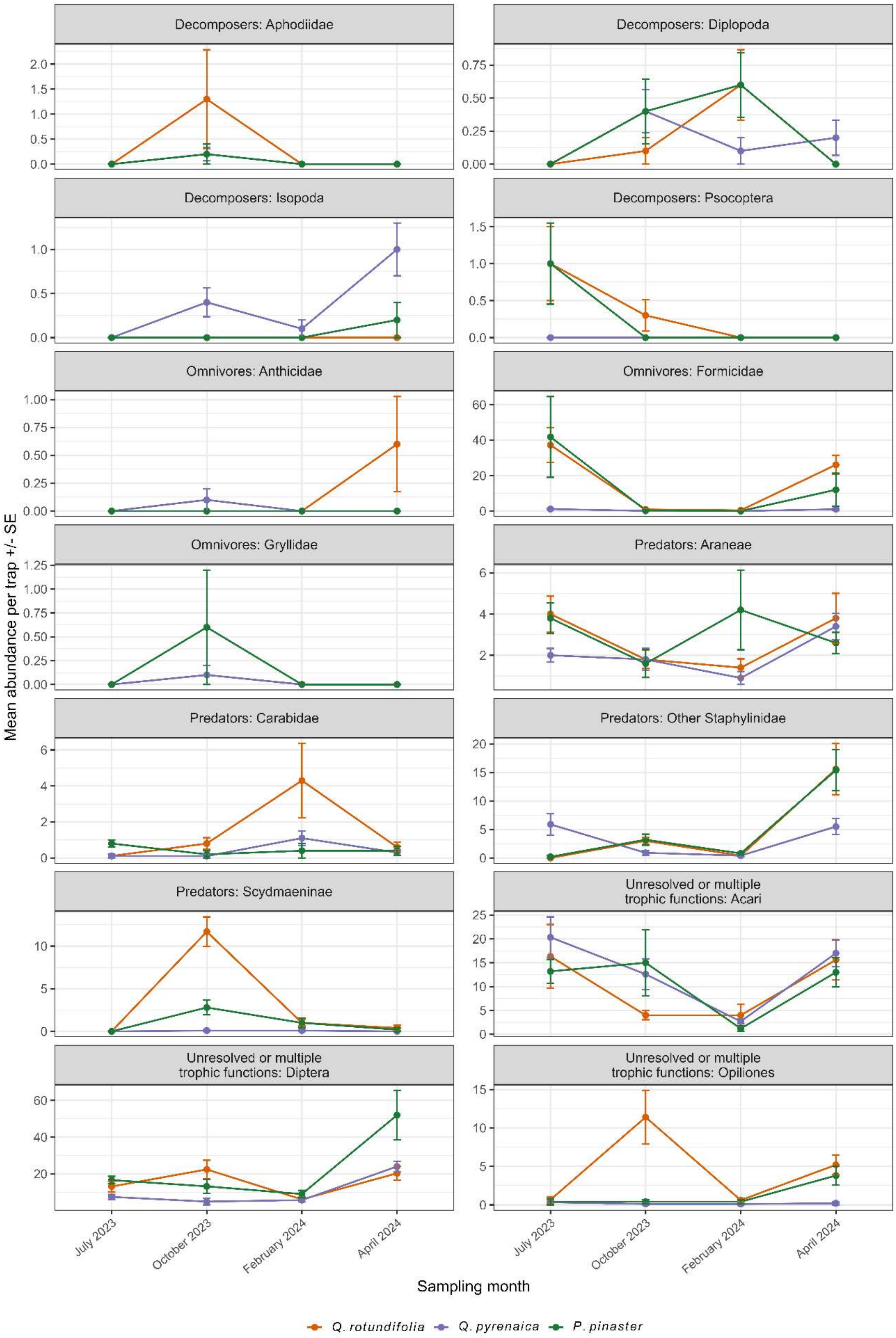
Decomposers: Aphodiidae
Decomposers: Diplopoda
Decomposers: Isopoda
Decomposers: Psocoptera
Omnivores: Anthicidae
Omnivores: Formicidae
Omnivores: Gryllidae
Predators: Araneae
Predators: Carabidae
Predators: Other Staphylinidae
Predators: Scydmaeninae
Unresolved or multiple trophic functions: Acari
Unresolved or multiple trophic functions: Diptera
Unresolved or multiple trophic functions: Opiliones
Mean abundance per trap +/- SE
July 2023
October 2023
February 2024
April 2024
Sampling month
Q. rotundifolia
Q. pyrenaica
P. pinaster

Figure S2. Temporal variation in the main taxa assigned to each functional group. Points represent mean abundance per trap and error bars represent standard errors of the mean.

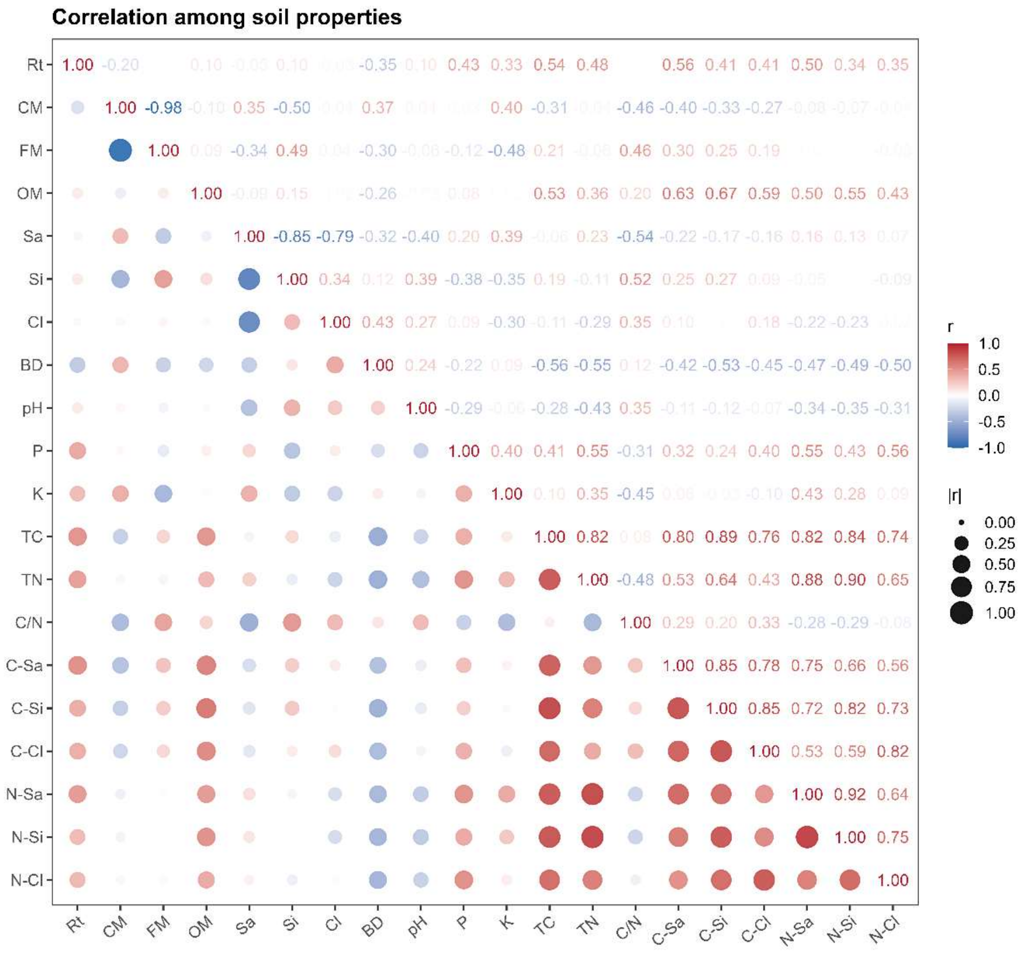

Figure S3. Pearson correlations among soil properties measured at 0–10 cm depth. The lower triangle shows correlations as circles, with circle size proportional to the absolute correlation coefficient (|r|), whereas the upper triangle displays the corresponding Pearson correlation coefficients. Red indicates positive correlations and blue indicates negative correlations; stronger colour intensity represents stronger associations. Rt, roots; CM, coarse material; FM, fine material; OM, organic matter; Sa, sand; Si, silt; Cl, clay; BD, bulk density; P, available phosphorus; K, available potassium; TC, total carbon; TN, total nitrogen; C/N, carbon-to-nitrogen ratio; C-Sa, carbon in the sand fraction; C-Si, carbon in the silt fraction; C-Cl, carbon in the clay fraction; N-Sa, nitrogen in the sand fraction; N-Si, nitrogen in the silt fraction; N-Cl, nitrogen in the clay fraction.
